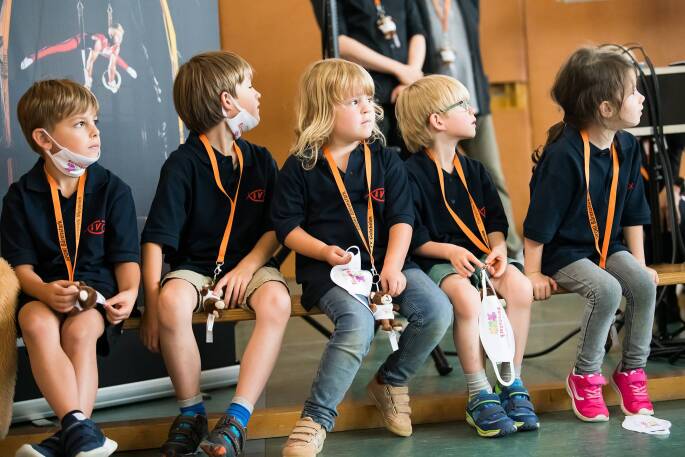# Gelingensbedingungen eines digitalen Sportangebots: Projektvorstellung

**DOI:** 10.1007/s43594-021-00030-z

**Published:** 2021-06-10

**Authors:** Julia Limmeroth, Norbert Hagemann, Florian Heussner, Volker Scheid

**Affiliations:** Institut für Sport und Sportwissenschaft, Universität Kassel, Kassel, Deutschland

**Welche Bausteine sind geeignet, um ein digitales Sportangebot für Kinder im Grundschulalter attraktiv zu gestalten? Das interdisziplinäre Projekt „Get Up **– **Stand Up** – **Move Up“ untersucht das.**

## Die Pandemie und der (Vereins‑)Sport

Seit Anfang des Jahres 2020 befindet sich die Welt in einer außergewöhnlichen, krisenhaften Situation. Die Menschen sehen sich mit der Covid‑19-Pandemie konfrontiert und in den meisten Ländern – auch in Deutschland – gelten Maßnahmen zur Kontaktbeschränkung. Dies hat auch für den (organisierten) Sport schwerwiegende Konsequenzen. Sportvereine – ausgenommen der Profisport – waren respektive sind weiterhin gezwungen, ihre Trainingseinheiten einzustellen beziehungsweise auf digitale Formate umzustellen. Damit einher gingen Saisonabbrüche in den Mannschaftssportarten. Allerdings blieb es vereinzelt möglich, zu zweit sportlich aktiv zu sein.

Mit Verlängerung des zweiten Lockdowns, der im November 2020 als Lockdown „light“ startete, spitzt sich auch die Situation für die Sportvereine zu. Besorgniserregend sind die Zahlen insbesondere bei Kindern und Jugendlichen (unter 18 Jahren). Dort verzeichnete beispielsweise der Landessportbund Hessen zwischen dem 1. Januar 2020 und dem 1. Januar 2021 einen Mitgliederrückgang um 6,8 %. Für Hessen bedeutet das, dass fast 63 % aller Mitgliederverluste auf Kinder und Jugendliche bis 18 Jahre zurückzuführen sind (Landessportbund Hessen 2021). Besonders betroffen sind Sportarten, die nicht draußen stattfinden können, sowie generell Kontaktsportarten.

Es zeigte sich darüber hinaus, dass zwei Drittel der Heranwachsenden in Deutschland besonders stark unter den Belastungen der Covid‑19-Pandemie leiden. Sie weisen eine signifikant niedrigere gesundheitsbezogene Lebensqualität, mehr psychische Gesundheitsprobleme und höhere Angstzustände als vor der Pandemie auf (Ravens-Sieberer et al. 2021). Laut Guan et al. (2020), deren Studienergebnisse sich auf 15 verschiedene Länder beziehen, zeigt sich, dass Kinder während der Schul- und Vereinsschließungen weniger körperlich aktiv waren, mehr gesessen und schlechter geschlafen haben im Vergleich zu der Zeit vor der Pandemie. Grundsätzlich lassen die Befunde und Trends darauf schließen, dass sich die immensen Einschränkungen des Sport- und Bewegungsangebots auf die Kinder und Jugendlichen und deren Aktivitätsverhalten nachhaltig negativ auswirken werden. In diesem Zusammenhang wird im Vierten Deutschen Kinder- und Jugendsportbericht unter anderem aufgezeigt, dass sportbezogenes soziales Lernen während des ersten Lockdowns kaum möglich war. Außerdem wurde die generelle Sozialisation zu Sport und Bewegung beeinträchtigt, was die Bindung von Kindern und Jugendlichen an die Institution Sportverein mit einbezieht (Breuer et al. 2020).

## Das Digitale und der Sport

**Figure 1:**
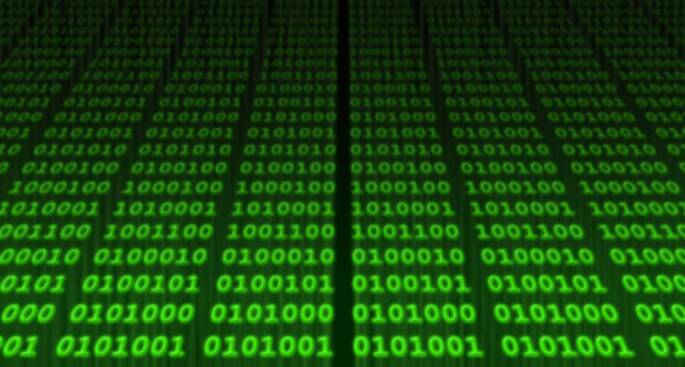

Unbestritten treibt die Covid‑19-Pandemie den digitalen Wandel rasant voran. Die Gesellschaft ist im Kontext physischer Distanzierung mehr denn je aufgefordert, digital zu arbeiten und zu kommunizieren sowie soziale Kontakte digital zu organisieren (Abb. 1). Bereits zuvor hat die Digitalisierung die bekannten Kulturtechniken durchdrungen und ist im Begriff, diese weiter zu verändern (Kerres 2017). Dadurch wird zwangsläufig Einfluss auf sämtliche Bereiche informeller, aber auch institutionalisierter Bildung genommen (Greve et al. 2020).

Im Kontext des Sports existiert zum Beispiel eine große Anzahl von Videos zum Selbsttraining zu Hause, die vorwiegend auf ein reines Vor‑/Nachmachen setzen und mittels unterschiedlicher Kanäle angeboten werden. Sowohl kommerzielle Anbieter und Privatpersonen als auch Institutionen, wie zum Beispiel Sportfachverbände, stellen Videos online zur Verfügung. So bietet unter anderem der Deutsche Handballbund über seinen Youtube-Kanal Übungsvideos mit Übungen für Kinder an (Deutscher Handballbund 2021). In Bezug auf das generelle Nutzungsverhalten und die -häufigkeit ist erkennbar, dass unter anderem das Alter einen zentralen Einfluss hat. So nutzen junge Menschen zwischen 14 und 29 Jahren digitale Dienste überdurchschnittlich intensiv und oft. Rund 9 % der Bevölkerung über 14 Jahre verwendeten 2020 eine Gesundheits- oder Fitness-App mehrmals pro Woche. Der Anteil derer, die überhaupt eine solche App nutzen, hat sich im Jahr 2020 im Vergleich zum Vorjahr um rund 6 % auf 33 % erhöht. Auch für Kinder und Jugendliche wurden neue Angebote ins Leben gerufen. Unter anderem hat der Basketballklub Alba Berlin bereits im Frühjahr 2020 das Online-Programm „ALBAs tägliche Sportstunde“ initiiert (Alba Berlin Basketballteam GmbH 2021). Dort werden täglich drei Sportstunden in einem altersgerechten Format angeboten (zunächst für Kitakinder, im Anschluss für Grundschulkinder und schließlich für Jugendliche).

Die angeführten Beispiele zeigen, dass es zahlreiche Ansätze zum digitalen Sporttreiben gibt. Es mangelt jedoch an einer Diskussion der eingesetzten Konzepte sowie einer begründeten pädagogisch-didaktischen Ausrichtung, insbesondere für den Kinder- und Jugendsport. So bleibt zu bedenken, dass bei den Einheiten mit Apps oder Videostreams niemand vor Ort ist, um falsche Haltungen und Bewegungsausführungen zu korrigieren oder Hilfestellungen zu geben. Das bedeutet, dass grundsätzlich gewisse Kompetenzen, zum Beispiel hinsichtlich der eigenen Leistungseinschätzung, gegeben sein müssen, um ohne Anweisungen beziehungsweise ohne direktes Feedback zu trainieren (Mutz und Gerke 2020). Hinzu kommt, dass durch den vorwiegenden Einsatz asynchroner Angebote wie Livestreams, Videos oder auch Apps die wesentlichen Aspekte der wechselseitigen Interaktion und des sozialen Miteinanders, die im Analogen manifeste Bestandteile des Sporttreibens im Sportverein sind, vernachlässigt werden.

Darüber hinaus ist jedoch festzustellen, dass der Digitalisierung im Sport bisher ein relativ geringer Stellenwert zugesprochen wurde und wird (Morlang 2020). Das hat sehr unterschiedliche Gründe. Dabei könnte die derzeit vorherrschende Situation um die Covid‑19-Pandemie genutzt werden, um die Relevanz, die digitale Medien für junge Menschen bereits vor der Pandemie eingenommen hat (Wolfert und Pupeter 2018), aufzugreifen und deren Potenzial zu nutzen sowie in innovative Sportangebote einzubinden. Langfristig könnten dadurch unter anderem folgende Ziele angestrebt werden: 1. Die Technologie so einsetzen, dass die selbständige sportliche Bewegung individuell unterstützt wird. 2. Die Attraktivität der Angebote für Kinder und Jugendliche durch den zusätzlichen Einsatz digitaler Medien erhöhen. 3. Gelingensbedingungen für soziale Interaktionen im sportlichen Setting mittels digitaler Medien generieren. Kurzfristig gilt es aber auch, den Vereinssport durch digitale Angebote zu unterstützen, um einerseits überhaupt Kontakt halten zu können und somit stabile, soziale Beziehungen zu gewährleisten sowie andererseits der zunehmenden Bewegungsarmut von Kindern auch in Pandemiezeiten etwas entgegenzusetzen (siehe auch Finger et al. 2018). Vermutlich bedeutet die Covid‑19-Pandemie auch für den organisierten Sport, sich auf neue Formate einzulassen und digitale Bausteine langfristig in ein innovatives Angebotskonzept einzubauen.

## Das Projekt „Get Up – Stand Up – Move Up“

Das Projekt „Digitales Sportangebot für Kinder im Grundschulalter: Get Up – Stand Up – Move Up“ widmete sich den angesprochenen Herausforderungen in mehrfacher Weise. Die Kooperation verschiedener regionaler Akteure, des Instituts für Sport und Sportwissenschaft der Universität Kassel (Arbeitsbereiche „Psychologie und Gesellschaft“ sowie „Erziehung und Unterricht“), des Transfer- und Anwendungszentrums Sport in Kassel (TASK) sowie des Fördervereins Handballjugend der TSG Wilhelmshöhe e. V., möchte zum einen mittels eines Bewegungsprogramms einen gesellschaftlichen Beitrag in dieser Krisensituation leisten. Zum anderen liegt der Fokus darauf, den Sport- und Bewegungsausfall von Kindern im Alter von fünf bis zehn Jahren aufzugreifen, ihm entgegenzuwirken sowie das Programm entsprechend wissenschaftlich zu begleiten. Dabei standen sowohl das gemeinsame Bewegen als auch die soziale Interaktion, die Beziehungsarbeit sowie die Förderung und Befriedigung der psychologischen Grundbedürfnisse im Vordergrund (Deci und Ryan 1985).

### Die Organisation:

**Figure 2:**
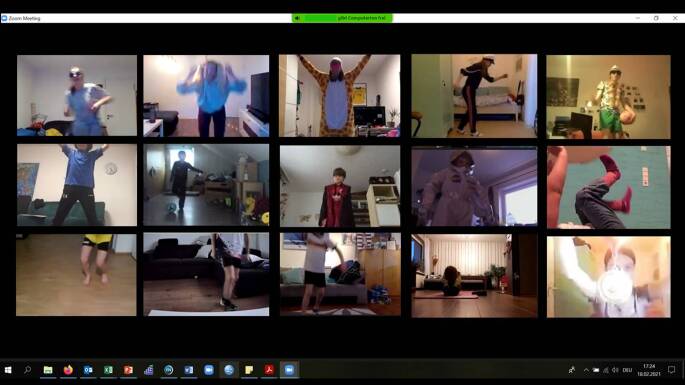

Zentrales Element des Projekts waren Videokonferenzen, die live stattfanden (Abb. 2). Das Projekt wurde mit 79 Kindern, aufgeteilt in acht Kleingruppen, gestartet. Die Tatsache, dass sich ein Großteil der Kinder sowie die Übungsleitenden vor der Teilnahme am Projekt nicht kannten und in neu zusammengestellten Gruppen agierten, stellte eine Besonderheit im Vergleich zu anderen digitalen Formaten dar. In diesem Sinne war es von besonderer Bedeutung, dass alle – Übungsleitende und Kinder – die Kamera sowie das Mikro angeschaltet hatten. Vorab wurden den Eltern Informationen zu den Übungsmaterialien per E‑Mail zugesendet. Jedes Kind war angehalten, den verfügbaren Raum so zu organisieren, dass es ausreichend Platz zum Bewegen hatte. Die 60 min dauernden Einheiten fanden zwischen Anfang Februar 2021 und Mitte Mai 2021 jede Woche statt.

Die Übungsstunden wurden über das Videokonferenzprogramm Zoom realisiert sowie von Sportstudierenden im Tandem mit Jugendlichen des Vereins durchgeführt und begleitet. Die ersten Minuten wurden stets dafür genutzt, die vergangene Woche aus einer sportlich-bewegungsbezogenen Perspektive zu reflektieren und sich auszutauschen. Den Anfang und das Ende einer jeden Einheit bildete ein Ritual, welches einem „virtuellen Körperklatschen“ entspricht. Angeleitet durch die Übungsleitenden wird dabei durch Klatschen in die Hände, auf die Brust und die Oberschenkel ein Rhythmus erzeugt und es werden gemeinsam verschiedene Sätze eingesprochen: „Wir – be-weg-en – uns – so – gern“ oder „Zu-sammen – be-weg-en – macht – uns – Spaß.“ Der entsprechende Rhythmus konnte von den Kindern mit neuen Ideen (Sätzen) weiterentwickelt werden. Vor dem Abschlussritual wurde die Einheit kurz reflektiert, zum Beispiel mit Hilfe der folgenden Fragen: „Welche Übung hat dir am meisten Spaß gemacht? Was wirst du später Mama/Papa zeigen?“

### Die inhaltliche Gestaltung:

Auf inhaltlicher Ebene wurden zunächst für diese Altersstufe zentrale Bewegungsfelder wie „Laufen, Springen, Werfen“, „Bewegen an und mit Geräten“, „Gymnastik, rhythmisches Bewegen, Tanzen“ sowie „Spielen“ in den Mittelpunkt gerückt. Neben vielfältigen Bewegungserfahrungen stand vor allem die Partizipation aller Kinder im Vordergrund. Die erste Einheit widmete sich dem Thema Laufen, Springen, Werfen („Schnell wie ein Reh: Olympiade zu Hause“), gefolgt von einer turnerischen Einheit, die entsprechende koordinative und konditionelle Fähigkeiten fokussierte („Turnen wie ein Äffchen: Das eigene Zuhause ganz neu erkunden“). Als dritte Einheit folgten ästhetische und gestalterische Formen von Bewegung („Bunt wie ein Chamäleon: Karneval des Sports zu Hause“). Die vierte Einheit fokussierte sich auf den Ball und entsprechende Einsatzmöglichkeiten in den eigenen vier Wänden („Ballspielfreudig wie ein Delfin: Der Ball muss nicht immer ins Eckige“). In den nachfolgenden Einheiten wurden die Schwerpunkte individuell auf die jeweiligen Gruppen angepasst, wobei Wünsche von Seiten der Kinder stets in die Planung mit einbezogen werden sollten. Neben der wöchentlichen Live-Einheit gab es ergänzende themenspezifische Übungen, die den Kindern über die Website des Fördervereins in Form von Bildreihen und PDF-Dokumenten zur Verfügung gestellt wurden. Dadurch sollte ein selbstgesteuertes, auf der Einheit aufbauendes Sportreiben – unabhängig vom Bildschirm – unterstützt werden.

Grundsätzlich waren alle Übungsleitenden angehalten, jedes Kind mindestens einmal pro Einheit individuell anzusprechen und mit einzubeziehen sowie Feedback zur Ausführung der Übungen zu geben und zu motivieren. Es wurden Formen der Mitbestimmung gewählt, die zum Beispiel dazu anregen, Spiele wie „Feuer, Wasser, Luft“ weiterzuentwickeln oder eigene Übungsideen zu integrieren (zum Beispiel bei „Ich packe meinen Koffer“). Durch die Arbeit im Tandem (Übungsleitende plus Jugendliche*r) konnten die Gruppen jeweils weiter aufgeteilt werden, sodass auch in kleineren Gruppen eigene Inhalte (zum Beispiel eine rhythmische Choreografie mit dem Ball) entwickelt werden konnten und die Kleingruppen sich gegenseitig etwas beibrachten. Darüber hinaus hat sich der Einsatz von Musik als überaus motivierend und spaßfördernd erwiesen. Es gilt hervorzuheben, dass es durch die Fokussierung auf die soziale Interaktion und die Förderung und Befriedigung der psychologischen Grundbedürfnisse unabdingbar war, mit Bild (Kamera an) sowie Ton (Mikro an) zu arbeiten. Das stellte für alle Beteiligten eine Herausforderung dar, hatte jedoch die gewünschte Interaktion und das Entstehen eines Gefühls des gemeinsamen Sporttreibens zur Folge. Insbesondere in dieser unmittelbaren und direkten digitalen Interaktion besteht der Mehrwert dieses Projekts. Darin besteht auch der Unterschied zu Angeboten, bei denen nur die Übungsleitenden im Bild zu sehen sind und die Teilnehmenden das Mikro aushaben, oder auch zu Videos, die asynchron eingesetzt werden.

Des Weiteren nimmt das Verhalten der Übungsleitenden einen zentralen Stellenwert ein und zielt darauf ab, soziale Eingebundenheit zu fördern sowie Kompetenz- und Autonomieerleben auf Seiten der Kinder zu ermöglichen (Ryan und Deci 2000). Der Spaß und die Freude an der Bewegung sollen vermittelt und „live“ vorgelebt werden. Insbesondere in der digitalen Anleitung sind die Übungsleitenden die zentralen Figuren, um positives Erleben auf Seiten der Kinder zu unterstützen. Durch ihr jeweiliges Verhalten und die Gestaltung der sportlichen Angebote werden die psychologischen Grundbedürfnisse angesprochen und beeinflusst. Positives Feedback, Ermutigungen, Anpassung des Schwierigkeitsgrads, wahrhaftiges Interesse und emotionale Qualität, um nur einige Aspekte zu nennen, wirken sich direkt auf das Erleben der Kinder aus. Es ist davon auszugehen, dass sich positive Zusammenhänge mit Freude und Vergnügen aufzeigen lassen, die auch in vorliegenden Studien gezeigt werden konnten (zum Beispiel White et al. 2018).

Bezugnehmend auf die Lebensphase der Kindheit treten in dieser zahlreiche Veränderungen in der Entwicklung zutage, die von einer stärker werdenden Differenzierung und Integrationsleistung geprägt sind. Dies zeigt sich sowohl im motorischen als auch im kognitiven Bereich. Ebenso werden Gefühle zunehmend ausdifferenzierter, und auch widersprüchliche Gefühlslagen müssen integriert werden. In diesem Sinne beschreibt Oerter (2008) mit Bezugnahme auf das Konzept der Entwicklungsaufgaben von Havighurst (1952), dass ab der Kindheit und besonders im Jugendalter die „peers“ zu bedeutenden Bezugspersonen avancieren. Der Entwicklung des Sozialverhaltens sowie des Verständnisses des eigenen Selbst kommt daher eine zentrale Aufgabe zu. Im Zentrum stehen die Ausbildung sozialer Kompetenzen sowie die Emotionsregulation, sodass in diesem Zusammenhang das Freundschaftsverständnis und die Entwicklung von Freundschaften in den Fokus der Betrachtung rücken. Das Spielen in Gruppen und das Arbeiten im Team sowie generell soziale Kooperationen müssen zunächst erlernt und entwickelt werden. Das gemeinsame Sporttreiben kann in diesem Sinne als ein zentraler Ort des sozialen Austausches und sozialen Miteinanders angesehen werden. Neben der für das ganze Leben zentralen Bedeutung eines körperlich aktiven Lebensstils ist es Sportvereinen wichtig, Werte wie ein soziales Miteinander oder Fairplay zu vermitteln, die im Einklang mit den entsprechenden Entwicklungsaufgaben des Kindesalters stehen (Breuer und Feiler 2019).

Darüber hinaus stellt das zivilgesellschaftliche Engagement in diesem Projekt für die jugendlichen Helfer*innen einen positiven Anker in krisenhaften Zeiten dar. Ihre Verantwortungsübernahme sollte sich auf zweifache Weise positiv auswirken: Erstens können sich die Jugendlichen als wirkungs- und bedeutungsvoll erfahren. Zweitens können sie durch das soziale Engagement ihre Bereitschaft und Fähigkeit zur Übernahme gesellschaftlicher Verantwortung stärken, wodurch ein Gefühl des Zusammenhalts und der Teilhabe entstehen kann (Düx et al. 2009). In Lockdown-Zeiten fiel diese Möglichkeit vielerorts weg oder war stark eingeschränkt. Buhl und Kuhn (2005) betonen die Bedeutung, die freiwilliges Engagement unabhängig von Familie, Freundeskreis oder Schule einnehmen kann und sehen darin die Möglichkeit, Erfahrungen zu sammeln, die für die Entwicklung des Selbstkonzepts, der moralischen Entwicklung sowie der gesellschaftlichen Verortung von grundlegender Bedeutung sind. In diesem Sinne wird mit dieser Art der Verantwortungsübernahme und des gesellschaftlichen Engagements sowohl ein persönlichkeitsbezogenes, individuelles als auch ein gesellschaftliches (Bildungs‑)Ziel auf dem Weg in das Erwachsenenalter verfolgt (Düx et al. 2009). Dies steht im Einklang mit den vier Entwicklungsaufgaben, die Hurrelmann und Quenzel (2016) für die Jugendphase unterscheiden: Qualifizieren, Binden, Konsumieren und Partizipieren. Diese beinhalten jeweils sowohl eine individuelle als auch eine gesellschaftliche Dimension. Letztlich setzt die Einbindung der Jugendlichen in das Projekt daran an und verfolgt das Ziel, dass sich die Jugendlichen durch die Vermittlungsaufgaben und Verantwortungsübernahme als selbstwirksam erfahren (Bandura 1986). Das heißt, dass sie durch das eigene Handeln wahrnehmen, ihre Lebenssituation wirkungsvoll beeinflussen zu können (Hurrelmann und Quenzel 2016).

## Ausblick

Das Projekt wird wissenschaftlich durch das Institut für Sport und Sportwissenschaft der Universität Kassel begleitet und evaluiert. Neben dem Ziel, Einblicke in die Gelingensbedingungen eines digital gestützten Sportangebots für Kinder zu erlangen, gilt das Interesse den Jugendlichen, die sich als Übungsleitende ehrenamtlich engagieren.

Auf Seiten der Kinder erfolgten zunächst eine Eingangsbefragung sowie eine weitere Befragung nach vier Wochen. Dabei lag der Fokus auf dem Sport- und Bewegungsverhalten der Kinder sowie der Förderung und Befriedigung der psychologischen Grundbedürfnisse. Diese Fragen beantworteten die Eltern und ihre Kinder in einer Online-Befragung (quantitativer Ansatz). Zusätzlich wurden vergleichend Kinder aus drei anderen Regionen Deutschlands, die nicht an dem Programm teilnahmen, befragt. In einem qualitativen Ansatz wurden sowohl einige Kinder als auch die studentischen Übungsleitenden in Form einer fokussierten beziehungsweise halbstrukturierten Interviewmethode befragt. Im Falle der Kinder kam die von Trautmann (2010) empfohlene, aber bisher noch selten angewandte Methode „Kinder interviewen Kinder“ zum Einsatz. Ziel der Interviews ist es, mehr über die Bedingungen, die zu einer positiven Beziehungsgestaltung auf digitaler Ebene im Kontext der sportlichen Aktivität führen, zu erfahren. Der Fokus liegt dabei auf der Fragestellung, ob das Projekt dazu beiträgt, psychologische Grundbedürfnisse zu fördern beziehungsweise zu befriedigen (Kohake und Lehnert 2018), und zu einem gesteigerten, selbstgesteuerten Sporttreiben führte.

Schließlich wurden die Jugendlichen, die im Rahmen des Projekts ehrenamtlich aktiv waren, interviewt, wodurch Einblicke hinsichtlich ihrer Perspektive auf ein verändertes soziales Engagement in Sportvereinen bedingt durch die Covid‑19-Pandemie gewonnen werden sollen (Kauer-Berk et al. 2020). Außerdem soll die Frage beleuchtet werden, ob ein derartiges Engagement die Einschätzung der Selbstwirksamkeit erhöhen kann.

Neben dem beschriebenen wissenschaftlichen Erkenntnisgewinn sollen die empirischen Daten dazu genutzt werden, das Programm bezogen auf die Inhalte und die digitale Umsetzung weiterzuentwickeln. Aspekte wie die Länge der Einheiten, die Art der Interaktion und die Stärken und Schwächen im Allgemeinen werden dabei in den Blick genommen. Die Beziehungsgestaltung in digitaler Form sollte weiterhin kritisch begleitet werden und die neuen digitalen Formate nicht als alleinige Formen des Sporttreibens aufgebaut werden. Mit Hilfe des Projekts „Get Up – Stand Up – Move Up“ soll vielmehr aufgezeigt werden, welche Bausteine geeignet sind, um ein ergänzendes digitales Sportangebot für Kinder attraktiv zu gestalten. Sei es, dass äußere Rahmenbedingungen reale Trainingseinheiten (Abb. 3) verhindern oder zukünftig innovative Angebotskonzepte aus realen und digitalen Formaten kombiniert werden, etwa um die Anleitung – über die in der Regel wöchentlichen Präsenztermine hinaus – in weiteren selbstverantworteten Übungseinheiten zu begleiten.

**Figure 3:**